# Establishment and Evaluation of Stable Cell Lines Inhibiting Foot-and-Mouth Disease Virus by RNA Interference

**DOI:** 10.1155/2014/109428

**Published:** 2014-02-10

**Authors:** Yuan-xing Gu, Zong-liang Gao, Jian-hua Zhou, Jie Zhang, Yong-sheng Liu

**Affiliations:** State Key Laboratory of Veterinary Etiological Biology, Lanzhou Veterinary Research Institute, Chinese Academy of Agricultural Sciences, Lanzhou, Gansu 730046, China

## Abstract

RNA interference (RNAi) has been proved to be a powerful tool for foot-and-mouth disease virus FMDV inhibition in vitro and in vivo. We established five stable baby hamster kidney 21 cell lines (BHK-21) containing five short hairpin RNAs (shRNAs) expression plasmids (p3D1shRNA, p3D2shRNA, p3D3shRNA, p3D4shRNA, and p3D5shRNA) targeting 3D gene of FMDV. Immunofluorescent assay, virus titration, and real-time quantitative reverse transcription polymerase chain reaction (Q-RT-PCR) were conducted to detect the effect of shRNAs on FMDV replication. After challenged with FMDV of O/CHA/99, two cell lines (p3D1shRNA and p3D4shRNA) showed a significant reduction in the synthesis of viral protein and RNA, accompanied by a sharp decrease in viral yield, and the inhibition could last for at least thirty passages. We developed an efficient procedure for the establishment and evaluation of stable cell lines for anti-FMDV research based on RNAi technology, which can be a candidate method for anti-FMDV research.

## 1. Introduction

Foot-and-mouth disease (FMD) is a highly contagious viral disease in cloven-hoofed animals caused by foot-and-mouth disease virus (FMDV). FMDV belongs to the *Aphthovirus* genus of the *Picornaviridae* family, and there are at least seven serological types, O, A, C, Asia1, SAT1, SAT2, and SAT3, and multiple subtypes [1, 2]. The FMDV genome is a single-stranded, positive sense, and linear molecule, about 8500 nucleotides in length, including a large single open reading frame (ORF) in the middle flanked by highly structured 5′ and 3′ untranslated regions. The single ORF encodes a polypeptide, which can be cleaved into the structural proteins (VP0/VP4, VP2, VP3, and VP1) and the nonstructural proteins (Lab, Lb, 2A, 2B, 2C, 3A, 3B, 3C, and 3D) [3, 4]. Particularly, the 3D gene is 1410 nucleotides in length and encodes a 470-amino-acid protein named viral RNA-dependent RNA polymerase (RDRP), which plays a central role in both transcription and viral genome replication, responsible for generating minus- and plus-sense genomic RNA [5]. Briefly, RDRP elongates a primer to copy the viral RNA template (plus strand) and the newly synthesized minus strand folds back on itself to generate a template-primer structure, which is elongated by the RDRP to form covalently linked dimeric RNA molecules [6, 7]. Due to its significance in viral replication, the 3D gene is highly conserved, especially within the functional motifs [8]. Comparative genomics of FMDV has revealed that the 3D protein is one of the most conserved FMDV proteins and proposed to be an ideal target for virus detection and classification; moreover, the RDRP may be more appropriate for phylogenetic analysis of the positive-strand RNA viruses than the capsid protein [4, 8, 9]. Previous study has demonstrated that the replication of poliovirus, which is also a member of the *Picornaviridae* family, can be strongly inhibited by RNAi targeted to the 3D gene [10]. So the 3D gene is an ideal potential RNAi target in anti-FMDV study. Antiviral strategies based on RNA interference (RNAi) have been documented for numerous viruses characterized by rapidity, specificity, and high efficiency [11]. Over the past decade, anti-FMDV has also focused on the RNAi technology, which targeted host genes that are responsible for viral replication such as *RNA helicase A *and *ectonucleoside triphosphate diphosphohydrolase 6 *[12, 13] and nearly all the regions of FMDV genome. However, the mechanism that determines the antiviral efficiency of RNAi in mammalian cells has not been completely defined.

Additionally, the duration, quantification, and side effect of RNAi-based antiviral strategy have not been well investigated, so the stable cell lines containing exogenous small interference RNAs (siRNAs) or short hairpin RNAs (shRNAs) are necessary to explore these questions. The procedure for generating stable cell lines by retroviral vectors or lentiviral vectors is complicated with a stringent specification for the materials and equipments. We established five stable baby hamster kidney 21 (BHK-21) cell lines containing different short hairpin RNAs (shRNAs) expression plasmids targeting the 3D gene of FMDV, which is a relatively convenient method to establish stable cells by RNAi.

## 2. Materials and Methods

### 2.1. Virus, Cell, and Vector

Baby hamster kidney cells 21 (BHK-21) were grown in Dulbecco's Modified Eagle's Medium (DMEM) supplemented with 10% fetal bovine serum (FBS; PH 7.4) in a humidified chamber at 37°C with 5% carbon dioxide (CO_2_). The pSilencer 4.1-CMV puro DNA vector (Ambion Inc., USA) employs a CMV promoter to drive high level expression of cloned hairpin siRNA, and it contains a puromycin antibiotic resistance marker which makes it conveniently to select for transfected cells. The culture medium supplemented with puromycin at 4 *μ*g/mL was used to select transfected cells. FMDV strain of O/CHA/99 was used for viral challenge [14].

### 2.2. Design of Hairpin siRNA

The sequence of O/CHA/99 (GenBank no. AF506822) was used as the RNAi template, and a web-based siRNA target finder was used for the calculation of potential RNAi targets (http://www.ambion.com/techlib/misc/siRNA_finder.html). Five different targets in 3D gene of FMDV and the negative control (pshRNA) which showed no homology to FMDV were picked out. All the selected targets and the corresponding shRNA templates were summarized in Table 1. Each group of the synthetic single strain DNA of the inverted repeat FMDV target sequences were annealed into a double strain DNA and cloned into the pSilencer 4.1-CMV puro vector (Ambion, Austin, USA) under the control of a powerful CMV promoter. The recombinant vectors were amplified through DH5*α* competent *Escherichia coli* cells and plasmid DNA extraction was conducted by the Endotoxin Free Plasmid Kit (Invitrogen, Carlsbad, USA). The recombinant vectors were confirmed by restriction enzyme digestion and sequencing.

### 2.3. Selecting Cells That Stably Express the shRNA

To create a cell clone expressing the shRNA stably, the recombinant vectors were transfected into BHK-21 cells by Lipofectamine 2000 reagent (Invitrogen, NY, USA), and the cells after transfection were grown in the medium supplemented with puromycin at 4 *μ*g/mL for approximately 14 days to eliminate the cells untransfected; then the macroscopic clones were picked out and continuously passaged in the medium supplemented with puromycin at 2 *μ*g/mL. PCR assay was employed to determine whether the transfected cells contain the recombinant vectors at the second, fifth, tenth, twentieth, and thirtieth passages. The primers for PCR assay were 5′-GGGATAACGCAGGAAAGA-3′ (forward) and 5′-GAACGACCTACACCGAAC T-3′ (reverse). The PCR products were analyzed on a 1.0% agarose gel.

### 2.4. Cell Toxicity Assay

To determine the viability of the transfected cells above, cell activity assay was carried out by CellTiter 96 AQ_ueous_ one solution (Promega, Madison, USA) according to the protocol. 1 × 10^4^ cells/well were seeded into a 96-well plate containing 100 *μ*L culture medium per well. After 24 h at 37°C, 5% CO_2_ atmosphere, 20 *μ*L/well of CellTiter 96 AQ_ueous_ one solution was added and incubated for 2 h; then the absorbance at 490 nm was recorded.

### 2.5. Analysis of FMDV Replication in Selected Cells

To detect the effect of siRNA on FMDV replication, indirect immunofluorescent assay (IFA), virus titrations, and quantitative real-time reverse transcription polymerase chain reaction (RT-PCR) were carried out.

#### 2.5.1. Indirect Immunofluorescent Assay

The indirect IFA was conducted as described previously [15]; briefly, BHK-21 cells were seeded on glass coverslips and grown to approximately 60% confluence and then infected with FMDV of O/CHA/99 at the multiplicity of infection (MOI) of 5. After 6 h after infection (hpi), cells were fixed with 3.7% paraformaldehyde, permeabilized with 0.2% Triton X-100, and blocked with 3% bovine serum albumin (BSA). Then cells were incubated in FMDV immune mouse ascites fluid (1 : 1000 dilution) at room temperature for 45 min, further reacted with a secondary goat-anti-mouse IgG antibody (1 : 100 dilution; Sigma, Saint Louis, USA), and conjugated with fluorescein isothiocyanate (FITC).

#### 2.5.2. Virus Titration Assay

A viral stock of titrated at 10^6.3^ 50% tissue culture infective dose (TCID_50_) per 0.1 mL was used for viral challenge. The transfected cells at the second, fifth, tenth, twentieth, and thirtieth passages in one well of the 96-well plates were infected with FMDV of 100TCID_50_ per 0.1 mL. The cells-lysed supernatant was collected at 24 h after infection, and viral titers were determined on BHK-21 cells three times and the TCID_50_ was calculated by the Reed-Muench method [16].

#### 2.5.3. Q-RT-PCR

To further detect the level of inhibition, the transfected cells at the second, fifth, tenth, twentieth, and thirtieth passages were challenged with FMDV at the MOI of 5 and the real-time Q-RT-PCR analysis was performed at 24 h after infection. Total RNA was extracted by Trizol reagent (Life Technologies, Carlsbad, USA), first incubated with RQ1 RNase-free DNase (Promega, Madison, USA) at 37°C for 30 min, then incubated with DNase stop solution at 65°C for 10 min, and then subjected to the real-time Q-RT-PCR analysis. The primers for the real-time Q-RT-PCR were 5′-GGGACCATACAGGAGAAGTT-3′ (forward) and 5′-CCCATCGCAGGTAAAGTG-3′(reverse). The real-time Q-RT-PCR program consisted of 95°C for 10 sec, followed by 40 cycles of 95°C for 5 sec and 60°C for 25 sec and then followed by 1 cycle of 95°C for 30 sec, 55°C for 30 sec, and 95°C for 20 sec at the dissociation stage. The real-time Q-RT-PCR analysis was performed on Stratagene real-time PCR Mx3000p (Agilent, Santa Clara, USA) using the SYBR* Premix Ex Taq* Kit (TaKaRa, Kyoto, Japan) according to the manufacturer's protocol. Melt-curve analysis of amplification products was performed at the end of each PCR reaction to confirm the specific amplification. The Q-RT-PCR products were analyzed on 1.0% agarose gel and further cloned into T-vector for sequencing. Reaction of each sample was performed in duplicate.

## 3. Results and Discussion

**Figure 1:**
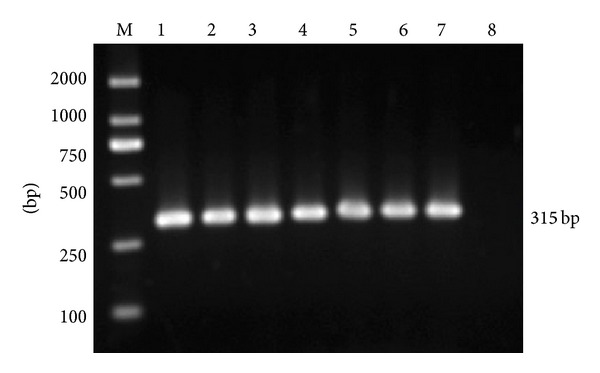
PCR products from transfected BHK-21 cells at the thirtieth passage were electrophoresed through 1.0% agarose gel. The expected PCR fragments were detected in lanes 1–8, which represents the pGAPDHshRNA, pshRNA, p3D1shRNA, p3D2shRNA, p3D3shRNA, p3D4shRNA, p3D5shRNA, and the mock, respectively.

**Figure 2:**
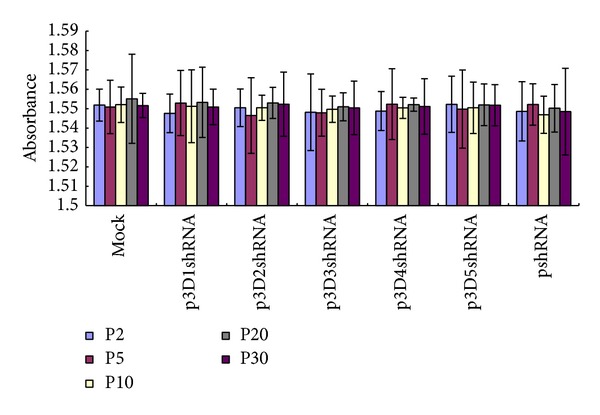
BHK-21 cells transfected with different recombinant vectors at the second, fifth, tenth, twentieth, and thirtieth passages were evaluated for the cytotoxicity of the recombinant vectors using CellTiter 96 AQ_ueous_ one solution. The absorbances at 490 nm were plotted using Microsoft Excel. There was no obvious difference between the different treatments and the mock (ANOVA test, *P* > 0.05). Error bars indicate standard deviations for three independent tests.

PCR products from transfected BHK-21 cells at the thirtieth passage was shown (Figure 1) and the sequencing results were correct. The results of the other passages detected were coincided with that of the thirtieth passage (data not shown), implying that the recombinant vectors were successfully introduced into the cells and replicated stably. According to the ANOVA test based on SPSS11.5 for Windows, no significant difference was observed between different treatments and the mock for all the passages detected (*P* > 0.05) (Figure 2), indicating that the recombinant vectors had no side effect on the transfected cells.

**Figure 3:**
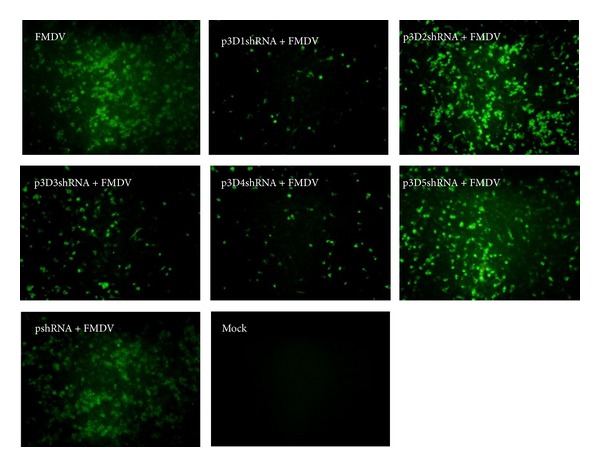
The results of the immunofluorescence assay for the cells transfected with the recombinant vectors at the thirtieth passage. The FMDV immune mouse ascites (1 : 1000) and a secondary goat-anti-mouse IgG antibody (1 : 100) conjugated with fluorescein isothiocyanate (FITC) were used to detect the viral protein. The specific fluorescence intensity under different recombinant vectors' treatments was lower than that of the mock and the negative control (pshRNA) at different levels.

The results of the IFA (the thirtieth passage) were shown in Figure 3, and the results of the second, fifth, tenth, and twentieth passages were well accordant with that of the thirtieth passage (data not shown). Generally, the fluorescence intensity of cells transfected with different recombinant vectors was lower than that of the cells transfected with pshRNA (pshRNA+FMDV) and the cells not transfected (FMDV). In detail, the fluorescence intensity of cells containing p3D1shRNA and p3D4shRNA reduced sharply compared with the other experimental groups, while the fluorescence intensity of cells containing p3D2shRNA, p3D3shRNA, and p3D5shRNA had slightly or even no decrease. The results indicated the shRNAs in our study could inhibit FMDV replication at different levels.

**Figure 4:**
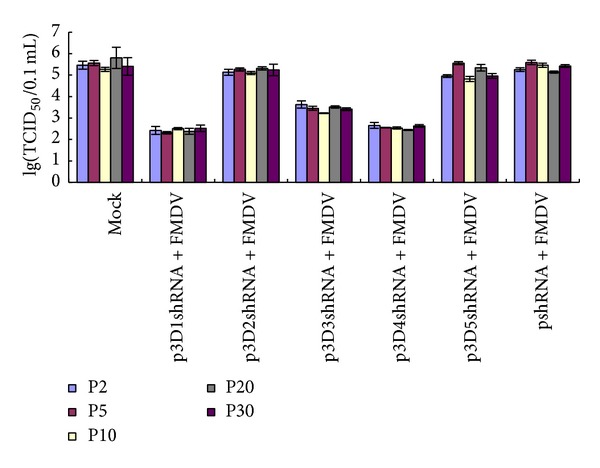
Decrease of viral yield in transfected cells with different recombinant vectors at the second, fifth, tenth, twentieth, and thirtieth passages. Cells transfected with the shRNAs expressing vectors were infected with FMDV O/CHA/99. Cells-lysed supernatant were collected at 24 h after infection, and the viral titers (TCID_50_) were determined on BHK-21 cells three times. The p3D1shRNA, p3D3shRNA, and p3D4shRNA showed a very significant difference compared with the pshRNA and the mock (ANOVA test, *P* < 0.001). Error bars indicate standard deviations.

Compared with the mock and negative control (pshRNA), the viral titers of cells transfected with different recombinant vectors were reduced at different levels (Figure 4). The mean value of the viral titers of all the passages detected decreased from 10^5.5^ TCID_50_ per 0.1 mL in mock to 10^2.4^, 10^5.2^, 10^3.5^, 10^2.6^, and 10^5.1^ TCID_50_ per 0.1 mL in cells transfected with p3D1shRNA, p3D2shRNA, p3D3shRNA, p3D4shRNA, and p3D5shRNA, respectively. The ANOVA test was carried out by the statistical software SPSS11.5 for Windows. The p3D1shRNA, p3D3shRNA, and p3D4shRNA showed a very significant difference compared with the pshRNA and the mock (*P* < 0.001) while p3D2shRNA and p3D5shRNA showed no significant difference compared with the pshRNA and the mock (*P* > 0.05). In addition, the same treatment among different passages had a similar inhibition effect on FMDV yield, suggesting that the recombinant vectors could maintain in the transfected cells stably.

**Figure 5:**
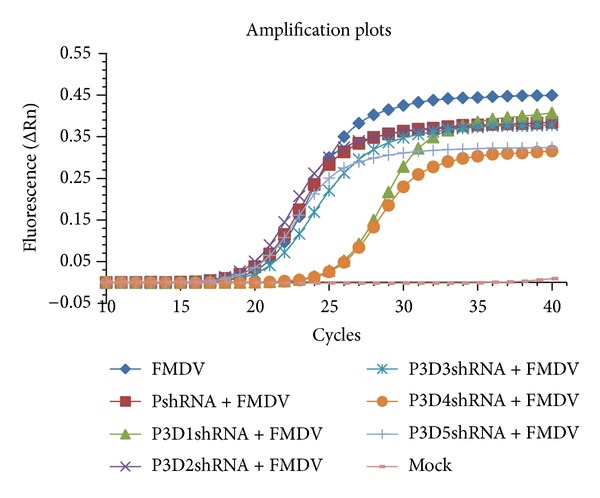
One amplification plot of real-time Q-RT-PCR analysis of the inhibition effects for the RNA replication of FMDV. Cells transfected with different expressing vectors at the second, fifth, tenth, twentieth, and thirtieth passages were infected with FMDV O/CHA/99. The real-time Q-RT-PCR analysis was performed on Stratagene real-time PCR Mx3000p system using the SYBR* Premix Ex Taq* Kit. Cells transfected with p3D1shRNA and p3D4shRNA showed an obviously lower level in RNA replication than the other groups.

The amplification plots of the real-time Q-RT-PCR at thirtieth passage were shown in Figure 5. Cells transfected with p3D1shRNA and p3D4shRNA showed an obviously lower level in RNA replication than the others groups. The mean cycle threshold (CT) values of p3D1shRNA and p3D4shRNA from two separate experiments were 26.24, and 26.23, respectively, while that of the other groups was about 21 (Table 2). The results of the real-time Q-RT-PCR at the second, fifth, tenth, and twentieth passages were similar to that of the thirtieth passage, suggesting that cells transfected with p3D1shRNA and p3D4shRNA could specifically inhibit the RNA replication of FMDV. Together with all the results in our study, we can draw a conclusion that two of the five targets (3D1 and 3D4) can strongly and stably inhibit FMDV replication in BHK-21 cells and all the recombinant vectors can sustain in BHK-21 cells stably.

Considerable literatures have grown up relating to experimental confirmation of the RNAi-based technology against FMDV since the first report on RNAi-mediated inhibition of FMDV [17]. However, not all RNAi targets can block virus replication effectively as previous research demonstrated. Transfection with shRNA expressing plasmids against the 1D gene of FMDV did not completely block virus replication in BHK-21 cells and the inhibition only lasted for 48 h, and identical results were obtained in IBRS-2 cells targeting the 2B gene [17, 18]. The results in present study also suggest that it is not easy to identify a completely effective target site for FMDV which includes so many variants, because both RNA structure and RNA-protein interaction can affect the function of RNAi. So it is a time consuming and laborious task to identify an effective RNAi target for the RNAi systems and further for the clinical applications. However, the procedure in our study supplies a relatively effective method to carry out long term study or cell target study about siRNA, which can be as a practical potential for the RNAi research against FMDV.

The majority of previous research on RNAi-based antiviral strategies against FMDV focused on the try of new vector systems and new target findings. Outstandingly, a multiple-shRNA expression system targeting different regions of FMDV genome showed broad antiviral effects against seven serotypes of FMDV [19]; better still, transgenic mouse models integrating siRNAs targeting 3D and 2B1 genes of FMDV have been established [20]. Despite great advances of RNAi as documented, many fundamental questions still have not been addressed. Only a few of factors that are likely to influence RNA silencing have been identified, and the “off-target” is clearly a problem for developing effective RNAi-based antiviral therapy [21]. Furthermore, no report has been available that the same target has an identical effect on the FMDV inhibition in different cell types or different serotypes of FMDV; namely, whether the RNAi target is cell-specific still undefined. In addition, there is no acknowledged standard to measure the quantitative relation between the exogenous siRNAs or shRNAs and the treatment effect in different cell types, and there is currently a lack of sufficient understanding that whether a memory mechanism of RNAi exists in mammalian cells. More experiments on the stable cell lines containing the exogenous siRNAs or shRNAs are needed to elucidate the details of RNAi in mammalian cells.

## 4. Conclusion

In present study, five stable BHK-21 cell lines expressing shRNAs targeting foot-and-mouth disease virus were established. Two of the established BHK-21 cell lines can block FMDV strongly. We developed an efficient procedure to identify RNA interference targets and to establish stable cell lines for FMDV inhibition by RNA interference. The cell stably expressing shRNA targeting FMDV can be used as a cell model for future study.

## Figures and Tables

**Table 1:** Target sequences of shRNA in 3D gene of FMDV used in this study.

Name	Target sequence (O/CHA/99, AF506822)	Oligonucleotide sequence
p3D1shRNA	5′-AAGACTCGCATTGTCGACGTC-3′ (nt 7196–7216 in the 3D region)	5′-GATCCGACTCGCATTGTCGACGTCTTCAAGAGAGACGTCGACAATGCGAGTCTTA-3′ (top strand) 5′-AGCTTAAGACTCGCATTGTCGACGTCTCTCTTGAAGACGTCGACAATGCGAGTCG-3′ (bottom strand)
p3D2shRNA	5′-AACAACATCTACGTGCTCTAC-3′ (nt 7598–7618 in the 3D region)	5′-GATCCCAACATCTACGTGCTCTACTTCAAGAGAGTAGAGCACGTAGATGTTGTTA-3′ (top strand) 5′-AGCTTAACAACATCTACGTGCTCTACTCTCTTGAAGTAGAGCACGTAGATGTTGG-3′′ (bottom strand)
p3D3shRNA	5′-AACATCTACGTGCTCTACGCT-3′ (nt 7601–7621 in the 3D region)	5′-GATCCCATCTACGTGCTCTACGCTTTCAAGAGA AGCGTAGAGCACGTAGATGTTA-3′ (top strand) 5′-AGCTTAACATCTACGTGCTCTACGCTTCTCTTGAAAGCGTAGAGCACGTAGATGG-3′′ (bottom strand)
p3D4shRNA	5′-AGACACTTCCACATGGACTA-3′ (nt 7828–7848 in the 3D region)	5′-GATCCGACACTTCCACATGGACTATTCAAGAGATAGTCCATGTGGAAGTGTCTTA-3′ (top strand) 5′-AGCTTAAGACACTTCCACATGGACTATCTCTTGAATAGTCCATGTGGAAGTGTCG-3′′ (bottom strand)
p3D5shRNA	5′-AAGTTACAGATCACTTTACCT-3′ (nt 8026–8046 in the 3D region)	5′-GATCCGTTACAGATCACTTTACCTTTCAAGAGA AGGTAAAGTGATCTGTAACTTA-3′ (top strand) 5′-AGCTTAAGTTACAGATCACTTTACCTTCTCTTGAAAGGTAAAGTGATCTGTAACG-3′′ (bottom strand)
pshRNA	5′-ACGGTGCCTTGCTCGCTAGAT-3′ (not homologous)	5′-GATCCGGTGCCTTGCTCGCTAGATTTCAAGAGAATCTAGCGAGCAAGGCACCGT A-3′ (top strand) 5′-AGCTTACGGTGCCTTGCTCGCTAGATTCTCTTGAAATCTAGCGAGCAAGGCACCG-3′ (bottom strand)

Sense and antisense sequence are underlined.

**Table 2:** The cycle threshold values (CT) of real-time Q-RT-PCR from the thirtieth passage.

Groups	CT range	CT mean
p3D1shRNA + FMDV	26.06–26.41	26.24
p3D2shRNA + FMDV	20.14–21.50	20.82
p3D3shRNA + FMDV	21.49–21.66	21.58
p3D4shRNA + FMDV	26.21–26.25	26.23
p3D5shRNA + FMDV	20.69–21.85	21.27
pshRNA + FMDV	20.61–21.62	21.12
FMDV	21.01–21.63	21.32
Mock	No CT	No CT
